# Clinical Outcomes and Therapeutic Response in Adult TFE3-Related Renal Cell Carcinoma: A Case Series From a Tertiary Cancer Center in Southern India

**DOI:** 10.7759/cureus.107915

**Published:** 2026-04-28

**Authors:** Kiran N Chavan, Lakshmi K Haridas, Neelima Radhakrishnan

**Affiliations:** 1 Medical Oncology, Regional Cancer Centre, Thiruvananthapuram, IND; 2 Pathology, Regional Cancer Centre, Thiruvananthapuram, IND

**Keywords:** adult renal carcinoma, immunohistochemistry, melanotic variant, targeted therapy, tfe3-rearranged rcc, xp11.2 translocation

## Abstract

TFE3-rearranged renal cell carcinoma (TFE3-RCC) is a rare molecular subtype of renal cell carcinoma (RCC), characterized by Xp11.2 translocations involving the *TFE3* gene. It is more common in pediatric populations and is often associated with aggressive clinical behavior and diagnostic challenges. Given the rarity of this entity in adults and limited therapeutic evidence, institutional case series remain valuable to enhance understanding of its clinicopathologic spectrum and outcomes. We conducted a retrospective case series of four adult female patients diagnosed with TFE3-rearranged RCC at a tertiary cancer center. Diagnosis was established based on morphology and strong nuclear TFE3 immunoreactivity, supported by additional immunohistochemical (IHC) markers including cluster of differentiation 10 (CD10), Melan-A, human melanoma black 45 (HMB45), vimentin, carbonic anhydrase IX (CAIX), and cytokeratins. Clinical presentation, radiologic findings, histopathologic features, immunohistochemical (IHC) profile, treatment modalities, and outcomes were reviewed. The median age was 32 years (range: 22-51). Two patients presented with localized stage III disease and underwent surgical resection (partial or radical nephrectomy), remaining disease-free at six months and one year of follow-up. Two patients presented with stage IV metastatic disease. One patient experienced late metastatic recurrence seven years after initial nephrectomy and is currently on cabozantinib with stable disease. The other had widespread metastases with spinal cord compression and showed disease progression despite targeted therapy and palliative radiotherapy, ultimately succumbing to the disease. Morphologically, tumors demonstrated heterogeneous features including clear cell, papillary, epithelioid, oncocytic, and melanotic differentiation. One case with coarse melanin pigmentation mimicked perivascular epithelioid cell tumor (PEComa), highlighting diagnostic complexity. TFE3-rearranged RCC in adults demonstrates heterogeneous morphology and variable clinical behavior, ranging from favorable outcomes in localized disease to aggressive progression in metastatic settings. Accurate diagnosis requires a high index of suspicion and comprehensive IHC evaluation, particularly in tumors with atypical morphology or melanotic differentiation. Advanced disease shows unpredictable response to targeted therapy, underscoring the need for further molecular characterization and prospective studies to optimize management strategies. Long-term surveillance remains essential due to the risk of late recurrence.

## Introduction

Renal cell carcinoma (RCC) accounts for approximately 2%-3% of adult malignancies worldwide, with nearly one-third of patients presenting with metastatic disease at diagnosis [1]. RCC represents a biologically heterogeneous group of tumors defined by distinct molecular drivers and clinicopathologic features [2]. Translocation-associated RCC involving the microphthalmia-associated transcription factor (MiT) family constitutes a rare but clinically important subtype [3]. These tumors are driven predominantly by rearrangements of the *TFE3 *gene at Xp11.2, resulting in oncogenic fusion proteins that dysregulate transcriptional pathways [3-5]. Recognition of this entity evolved with molecular discoveries, leading to its formal inclusion as Xp11.2 translocation RCC in the 2004 World Health Organization (WHO) classification [1].

Subsequently, the 2016 WHO classification grouped TFE3-rearranged RCC with TFEB-altered tumors under the broader category of MiT family translocation RCC, reflecting shared biological and morphologic features [2]. The 2022 WHO Classification further refined renal tumor taxonomy by removing the term MiT family translocation RCC and recognizing TFE3-rearranged RCC and TFEB-altered RCC as two separate molecular entities, emphasizing the prognostic and diagnostic importance of accurate classification [6].

TFE3-rearranged RCC comprises up to 20%-40% of pediatric RCC but only 1%-4% of adult cases [7-9]. In adults, it is frequently associated with advanced stage at presentation, aggressive clinical behavior, and inferior outcomes compared with conventional RCC subtypes [10-12]. Diagnostic challenges arise from its morphologic overlap with clear cell RCC, papillary RCC, and perivascular epithelioid cell tumor (PEComa, a rare mesenchymal neoplasm with melanocytic differentiation), as well as rare variants such as melanotic differentiation [13]. Ge et al. [14] demonstrated that clinicopathologic behavior in Xp11.2 translocation renal cell carcinoma varies according to the specific TFE3 fusion partner, underscoring the prognostic and potential therapeutic relevance of molecular subtype stratification.

A retrospective study of 26 patients from India has shown that adult TFE3-rearranged RCC exhibits aggressive behavior, with 64% relapse after nephrectomy and a median progression time of 13 months. Overall survival was limited with median overall survival (OS) 30 months; 17 months with systemic therapy and vascular endothelial growth factor (VEGF)-targeted therapy showed only short-lived benefit (median event-free survival (EFS): 8 months first-line, 2.5 months second-line) [15]. Given its rarity, heterogeneity, and lack of standardized treatment guidelines, especially in adults, institutional case series remain valuable for improving recognition and understanding of this entity [10,11]. We present a case series of TFE3-related RCC highlighting clinicopathologic features, diagnostic pitfalls, and treatment outcomes, with emphasis on the evolving WHO classification and its clinical implications.

## Case presentation

Case 1

A 51-year-old woman was initially diagnosed in 2018 with stage III left renal cell carcinoma, underwent radical nephrectomy, and was reported as clear cell RCC. She subsequently received external beam radiotherapy (EBRT) and targeted therapy at an outside center; however, the indication, site, and dose of radiotherapy could not be ascertained due to the unavailability of prior treatment records, remaining clinically disease-free for approximately seven years.

**Figure 1:**
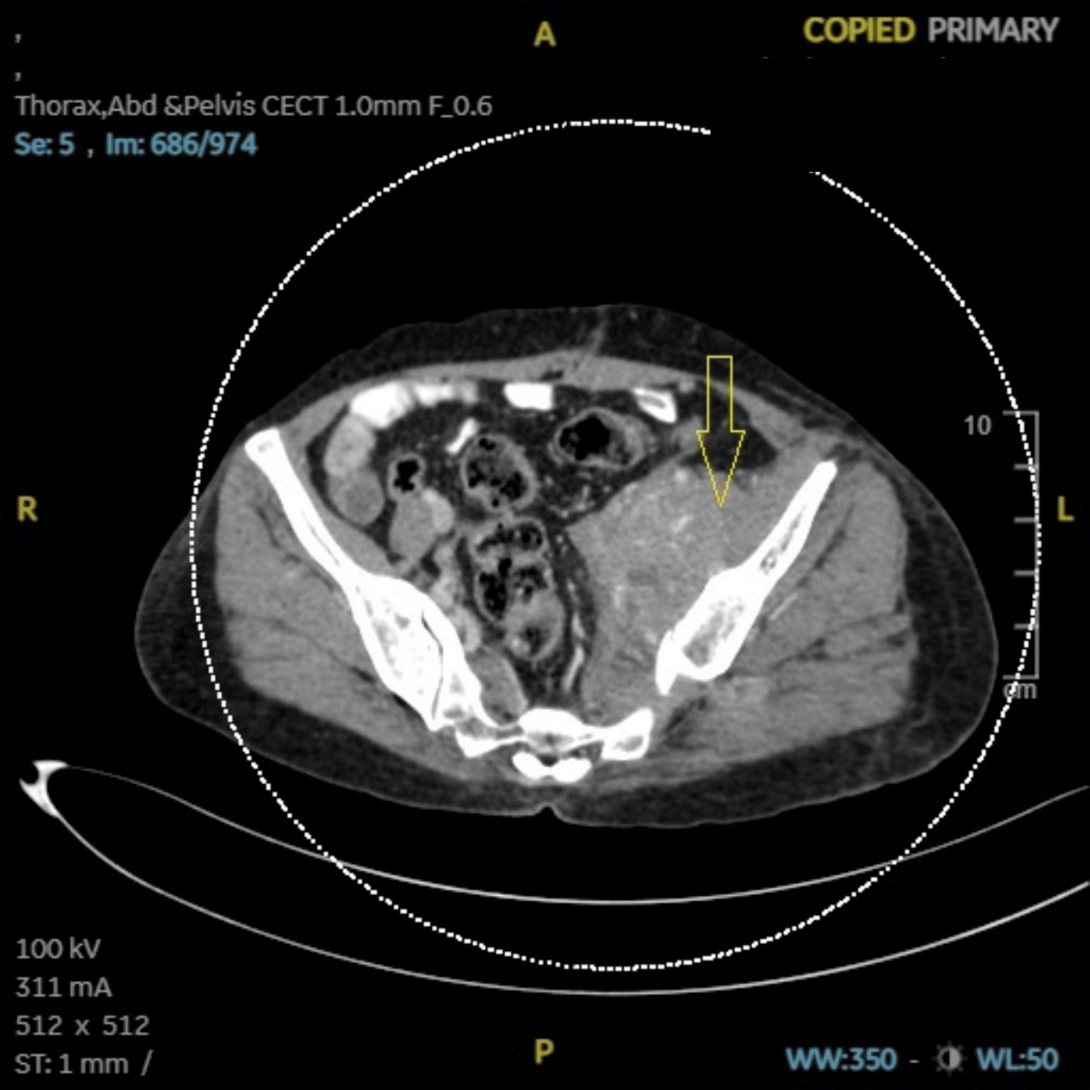
Case 1: CECT (abdomen and pelvis) showing left pelvic nodal mass (9 × 10 × 6.5 cm) along iliac vessels encasing left external iliac artery, infiltrating left iliopsoas, obturator internus muscle (arrow). CECT: contrast-enhanced computed tomography, A: anterior, R: right, L: left, P: posterior, ST: soft tissue, WW: window width, WL: window level.

**Figure 2:**
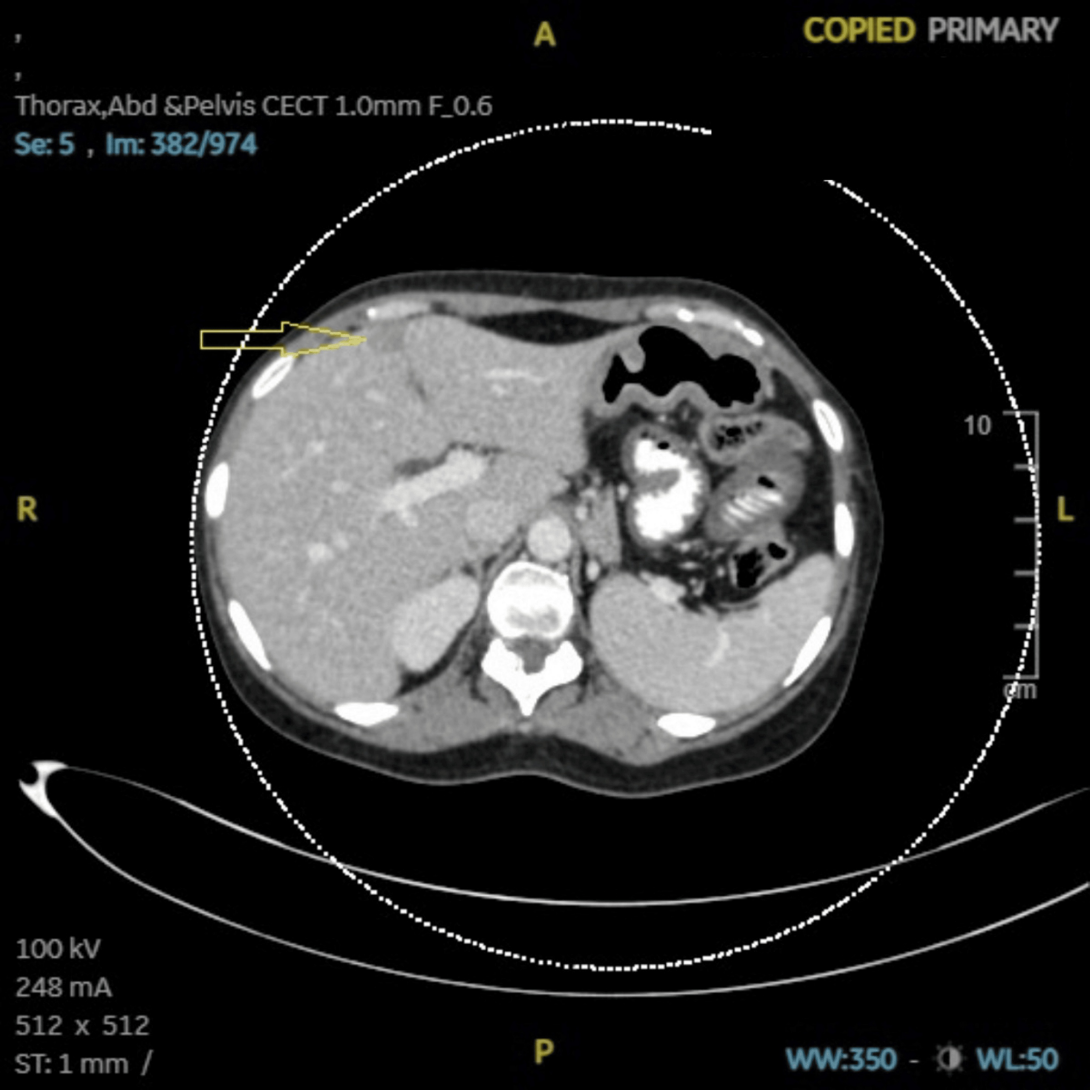
Case 1: CECT (abdomen and pelvis) showing hypoenhancing lesion (14 × 10 mm) in liver in sub-capsular parenchyma of segment IVB (arrow). CECT: contrast-enhanced computed tomography, A: anterior, R: right, L: left, P: posterior, ST: soft tissue, WW: window width, WL: window level, IVB: segment IVB of liver.

**Figure 3:**
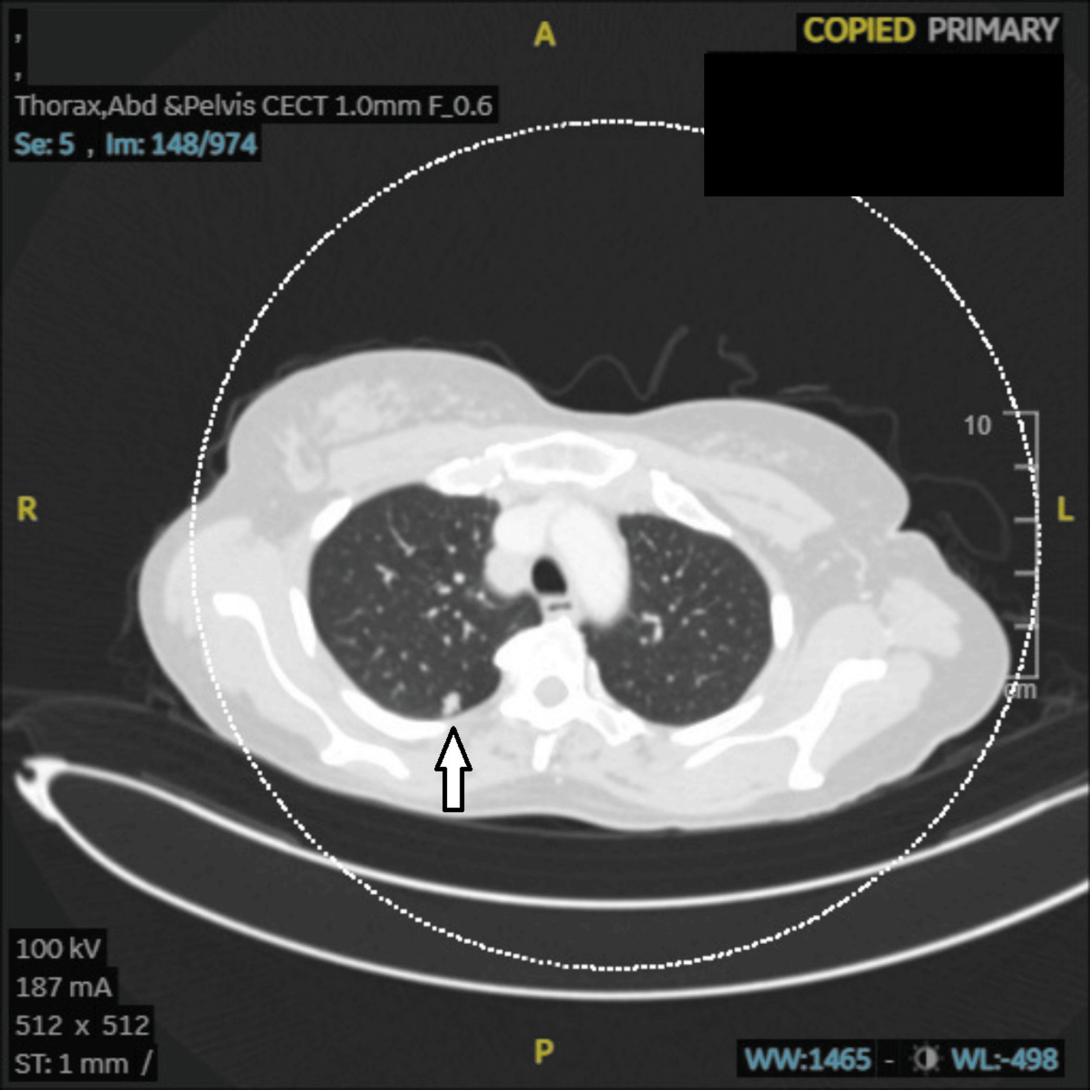
Case 1: CECT (chest) showing right upper lobe posterior segment, well-defined non-calcified nodule with lobular end margins (8.5 × 7 mm) likely benign (arrow). CECT: contrast-enhanced computed tomography, A: anterior, R: right, L: left, P: posterior, ST: soft tissue, WW: window width, WL: window level.

**Figure 4:**
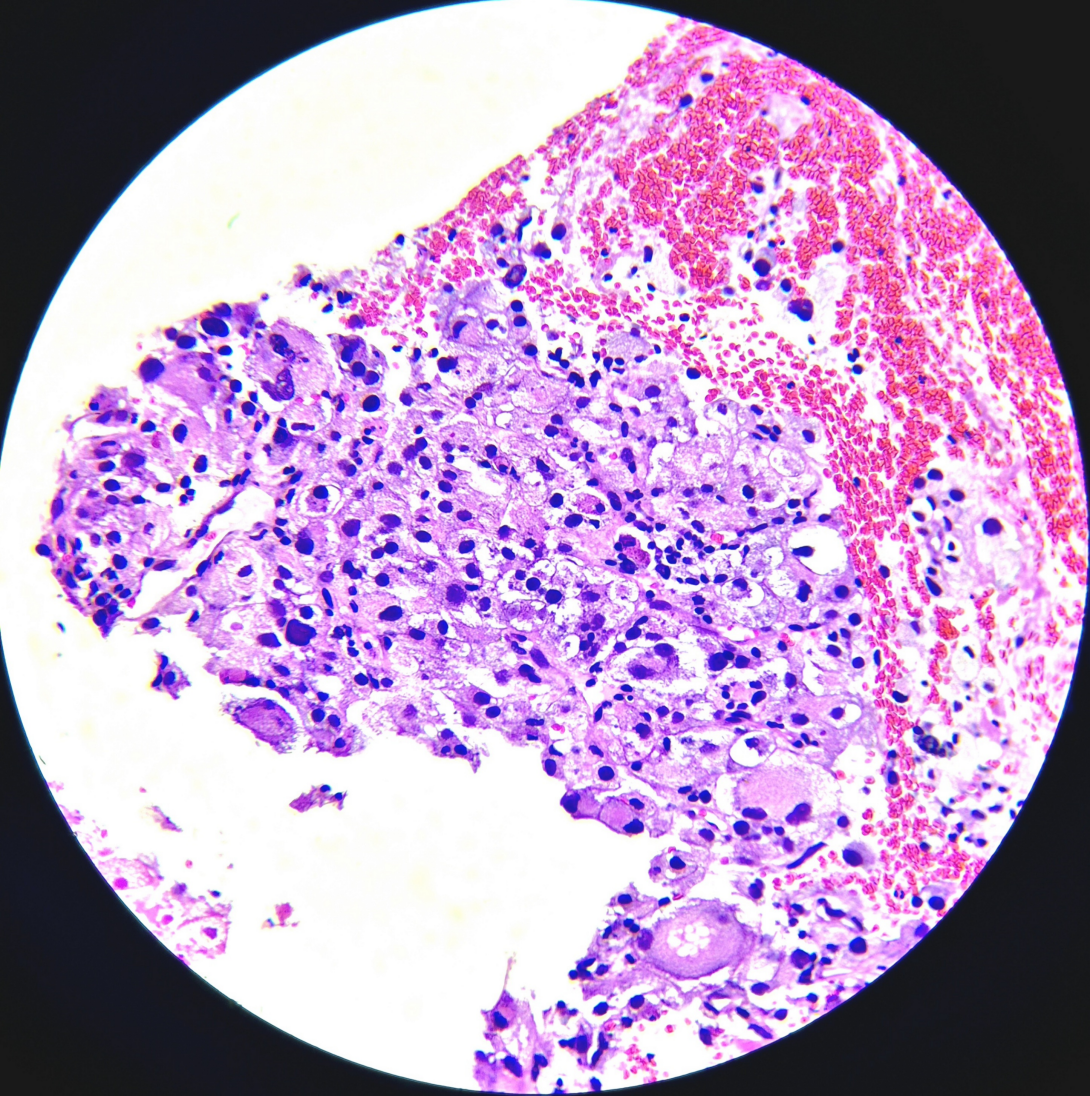
Case 1: Left inguinal lymph node biopsy HE section (40×) shows neoplastic cell predominantly arranged in sheets. The tumor cells are large and polygonal with round to oval hyperchromatic nuclei and abundant eosinophilic granular vacuolated cytoplasm. HE: hematoxylin and eosin stain.

**Figure 5:**
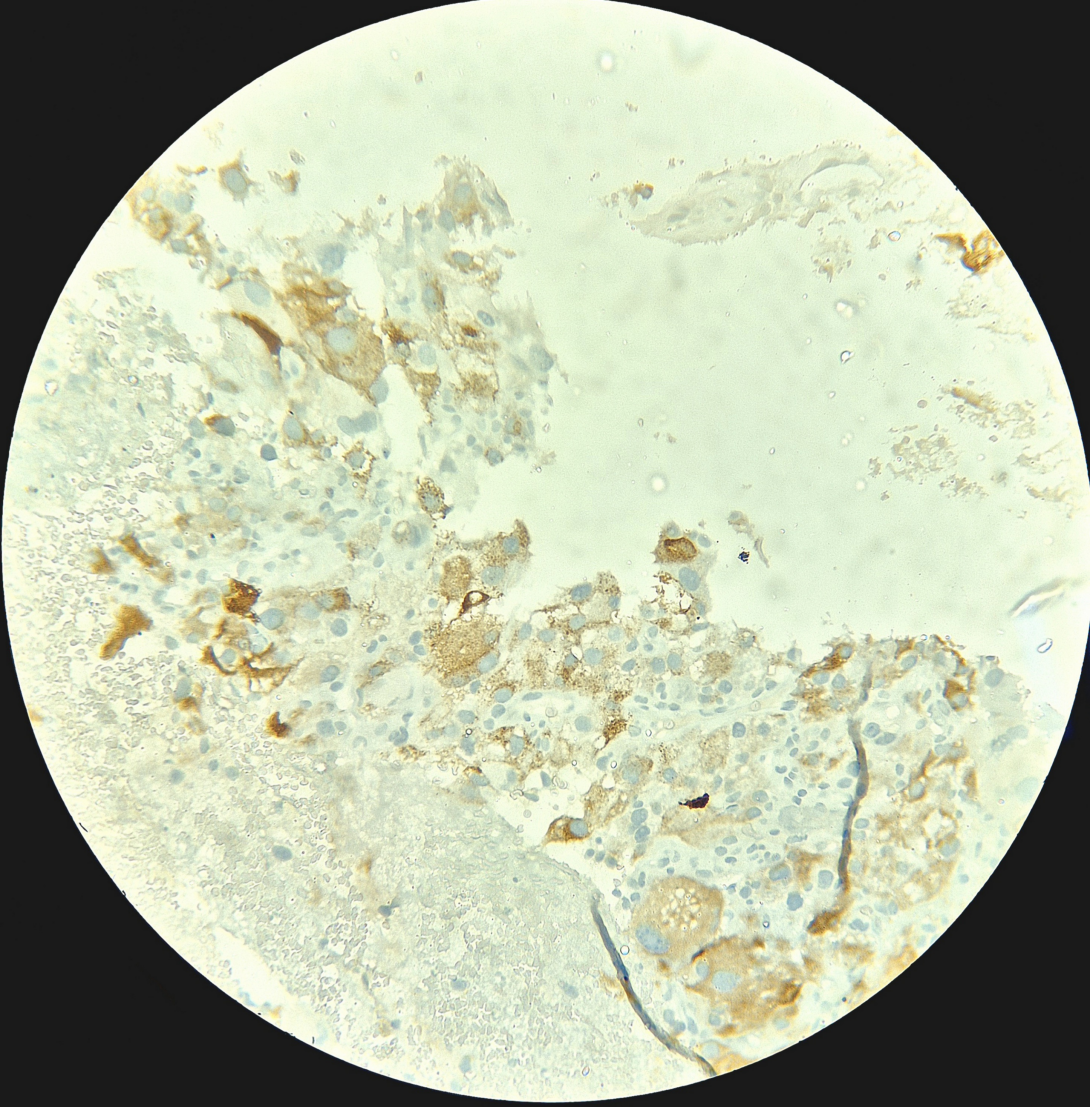
Case 1: IHC tumor cell shows diffuse cytoplasmic positivity for Melan-A. IHC: immunohistochemical.

**Figure 6:**
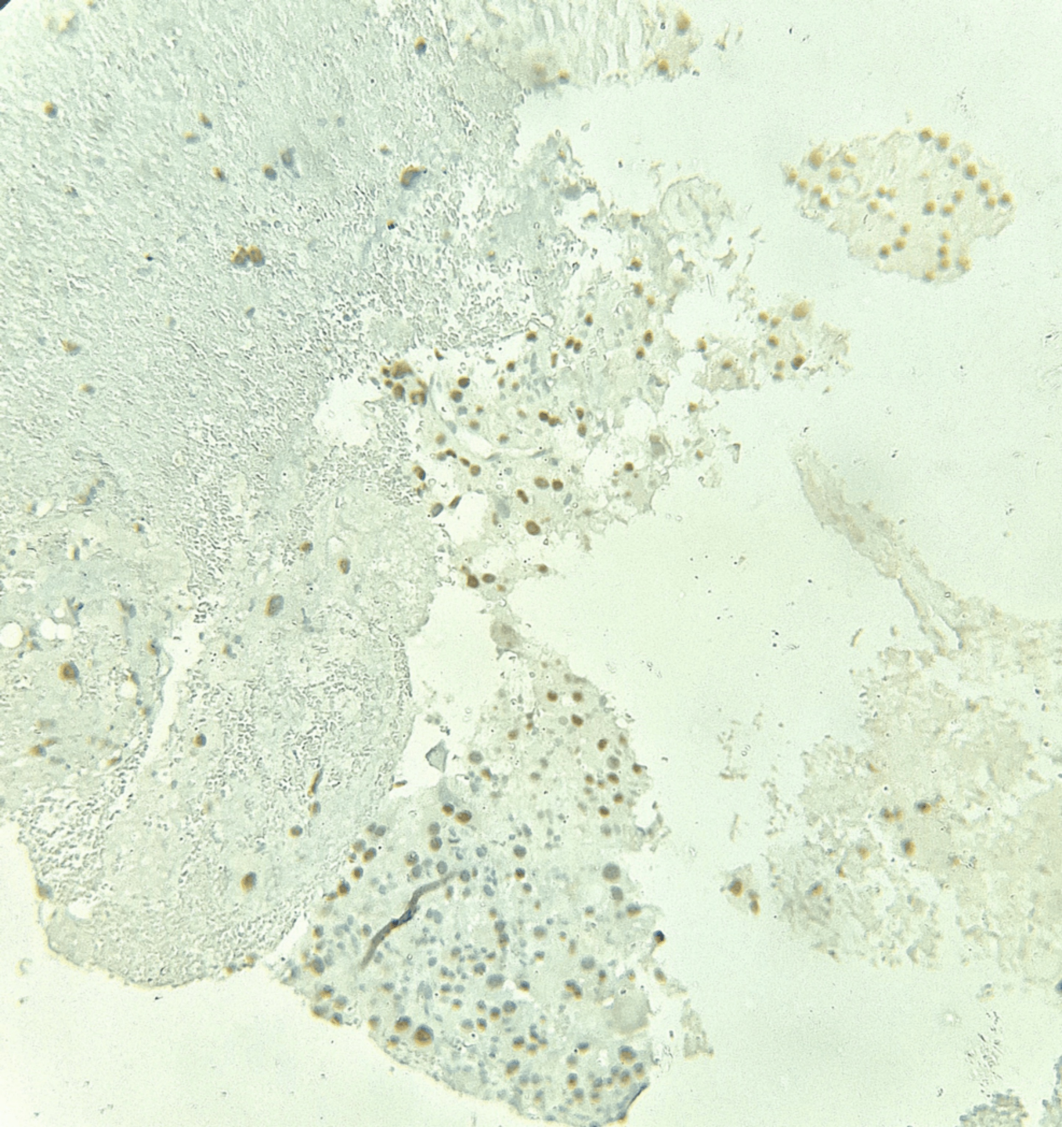
Case 1: IHC tumor cells shows nuclear positivity for TFE3. IHC: immunohistochemical.

In February 2025, she presented to our institute with symptoms related to abdominal and pelvic disease burden. Imaging revealed retroperitoneal and pelvic lymphadenopathy (Figure 1), a liver lesion measuring 14 × 10 mm (Figure 2), and an indeterminate 5 mm right lung nodule (Figure 3). Biopsy of a metastatic site demonstrated a metastatic malignant neoplasm with epithelioid, clear cell, and oncocytic morphology arranged in sheets; individual cells are large and polygonal with round to oval hyperchromatic nuclei and abundant eosinophilic, granular, vacuolated cytoplasm (Figure 4); and IHC showed negative cytokeratin expression and positive melanocytic markers (Melan-A) (Figure 5) and TFE3 (Figure 6) consistent with TFE3-rearranged RCC. She was diagnosed with TFE3-rearranged RCC stage IV with metastatic involvement of liver and abdominal and pelvic lymph nodes (International Metastatic RCC Database Consortium (IMDC) poor risk, based on anemia (hemoglobin: 7.2 g/dL), thrombocytosis (platelet count: 620,000/µL), and time from diagnosis to initiation of systemic therapy <1 year, with no additional risk factors including poor performance status (Karnofsky Performance Status (KPS): 90), hypercalcemia (corrected calcium: 9 mg/dL), or neutrophilia (absolute neutrophil count (ANC): 6,100/µL)), and started on Cabozantinib at a dose of 60 mg once daily from August 2025. The dose was subsequently reduced to 40 mg once daily based on tolerability. She is currently tolerating treatment well and remains clinically stable; response assessment imaging is awaited.

Case 2

**Figure 7:**
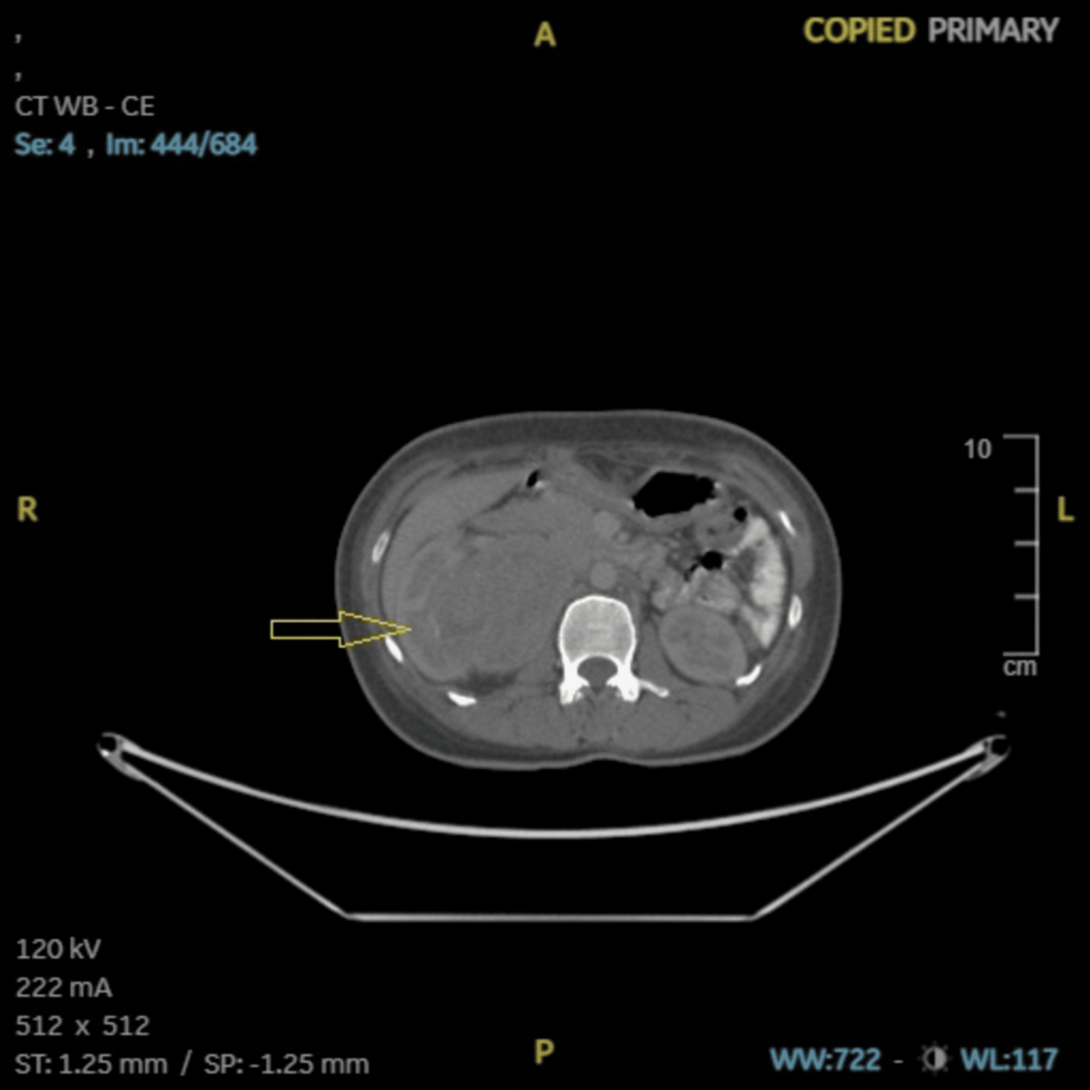
Case 2: CECT (abdomen and pelvis), a primary right renal mass measuring 6.5 × 5.5 × 6 cm (arrow). CECT: contrast-enhanced computed tomography, A: anterior, R: right, L: left, P: posterior, ST: soft tissue, SP: soft tissue window (preset), WB: whole body, WW: window width, WL: window level.

**Figure 8:**
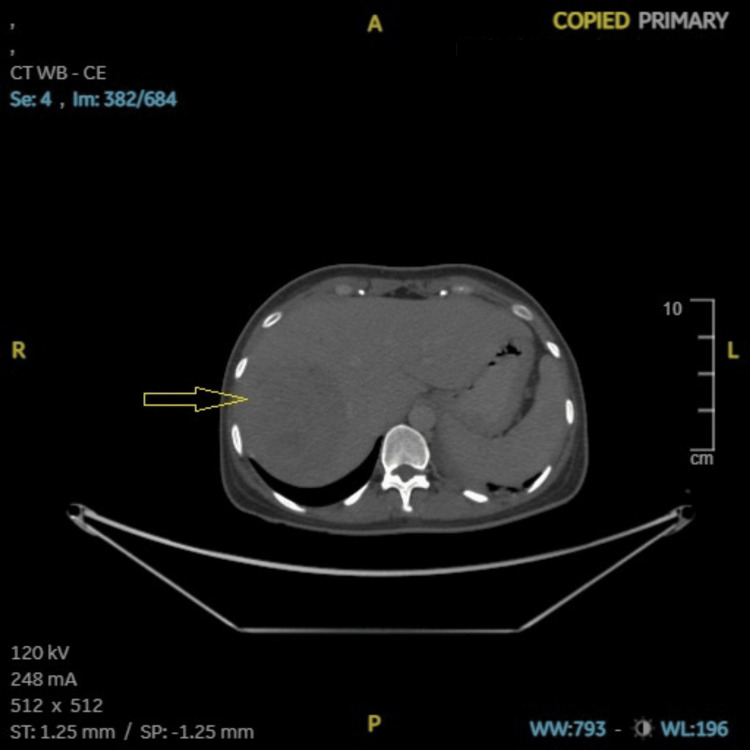
Case 2: CECT (abdomen and pelvis) showing hypoenhancing lesion in liver (arrow). CECT: contrast-enhanced computed tomography, A: anterior, R: right, L: left, P: posterior, ST: soft tissue, SP: soft tissue window (preset), WB: whole body, WW: window width, WL: window level.

**Figure 9:**
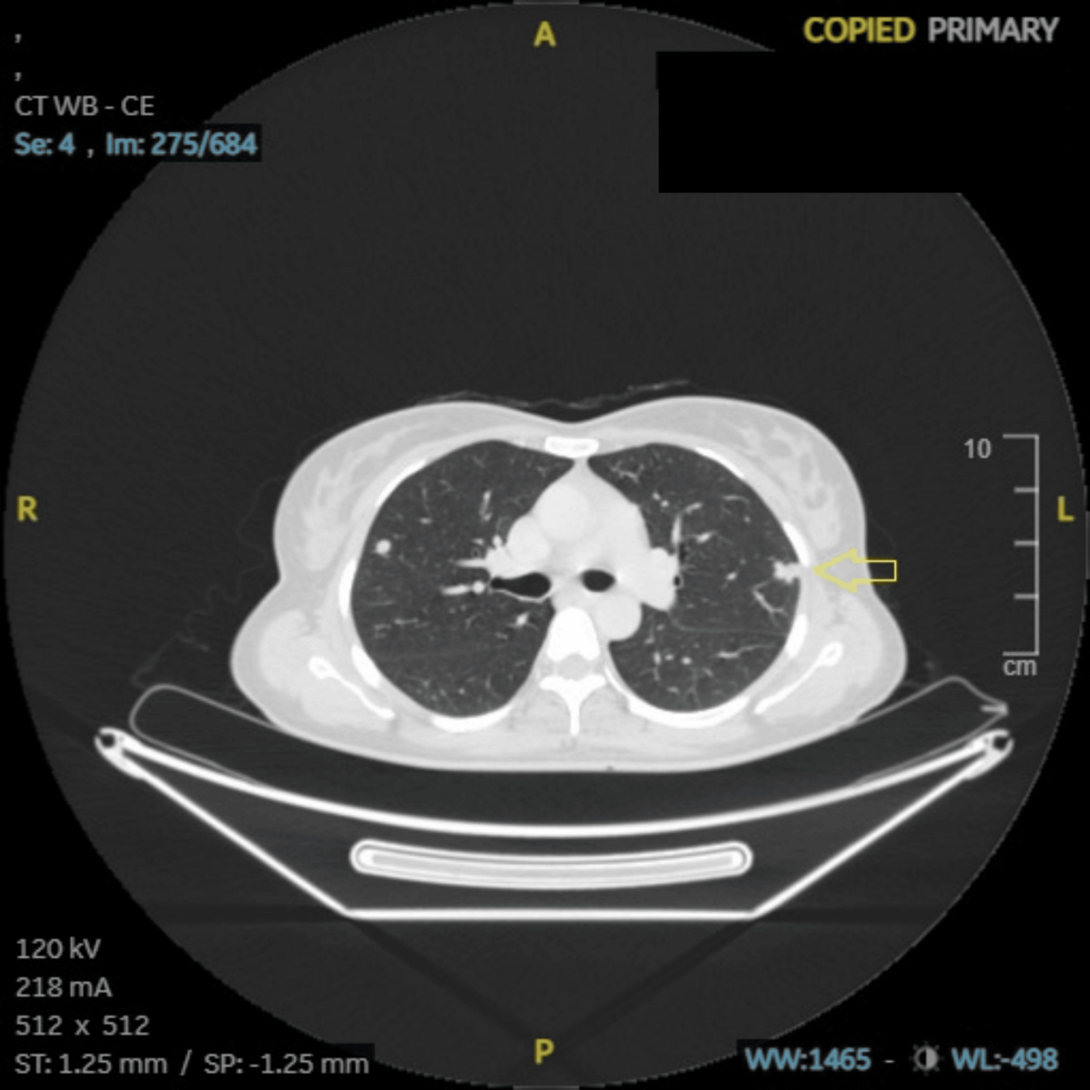
Case 2: CECT (abdomen and pelvis), soft tissue nodules in left lung upper lobe (6 × 4.5 cm), multiple soft tissue nodules in bilateral lung parenchyma (arrow). CECT: contrast-enhanced computed tomography, A: anterior, R: right, L: left, P: posterior, ST: soft tissue, SP: soft tissue window (preset), WB: whole body, WW: window width, WL: window level.

A 43-year-old woman presented with advanced non-clear renal cell carcinoma, stage IV, with a primary right renal mass measuring 6.5 × 5.5 × 6 cm (Figure 7) and multiple metastases involving the liver (Figure 8), lungs (Figure 9), adrenal gland, and bone.

**Figure 10:**
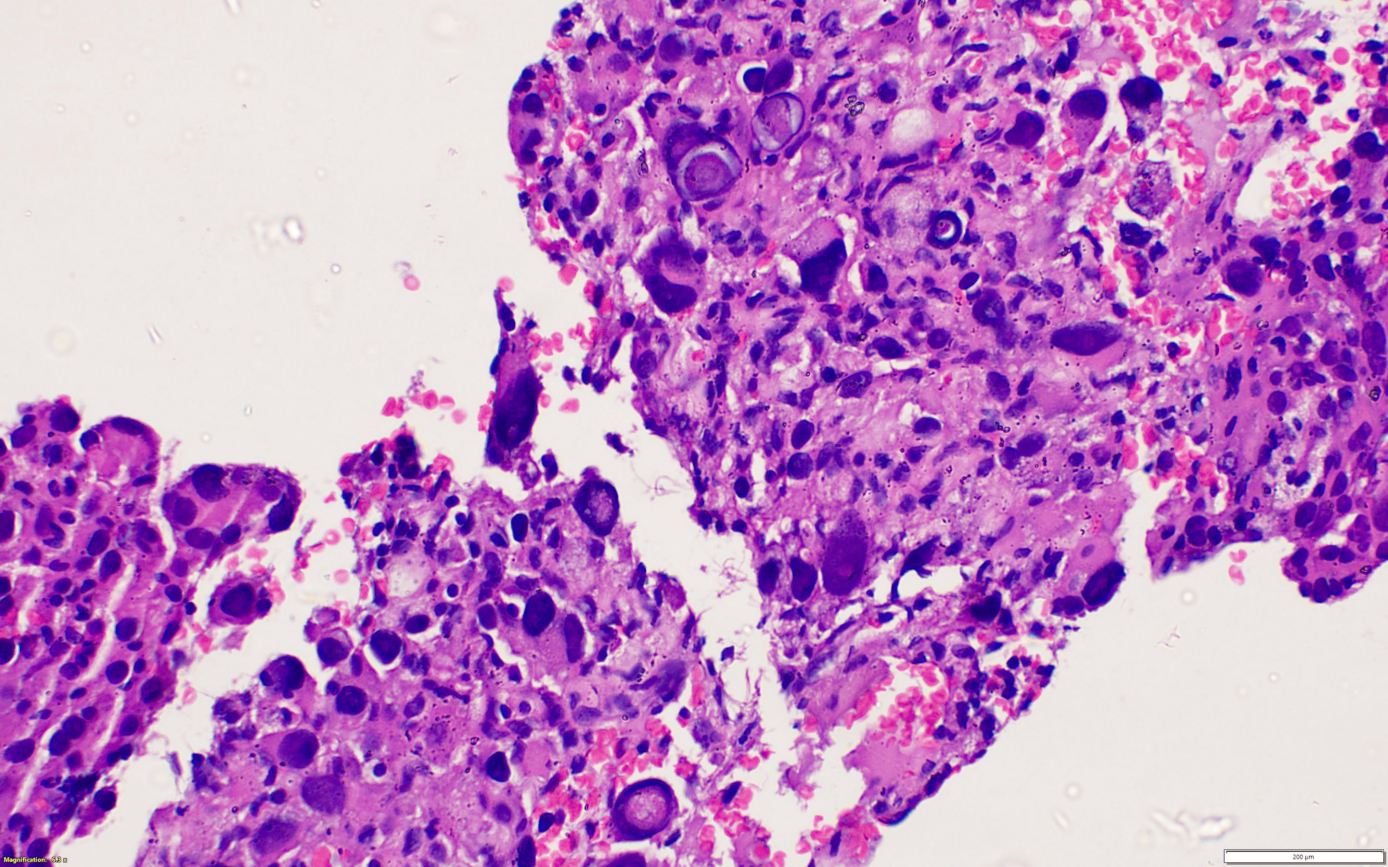
Case 2: Liver lesion biopsy (HE), high‑grade malignant neoplasm with epithelial features. HE: hematoxylin and eosin stain.

**Figure 11:**
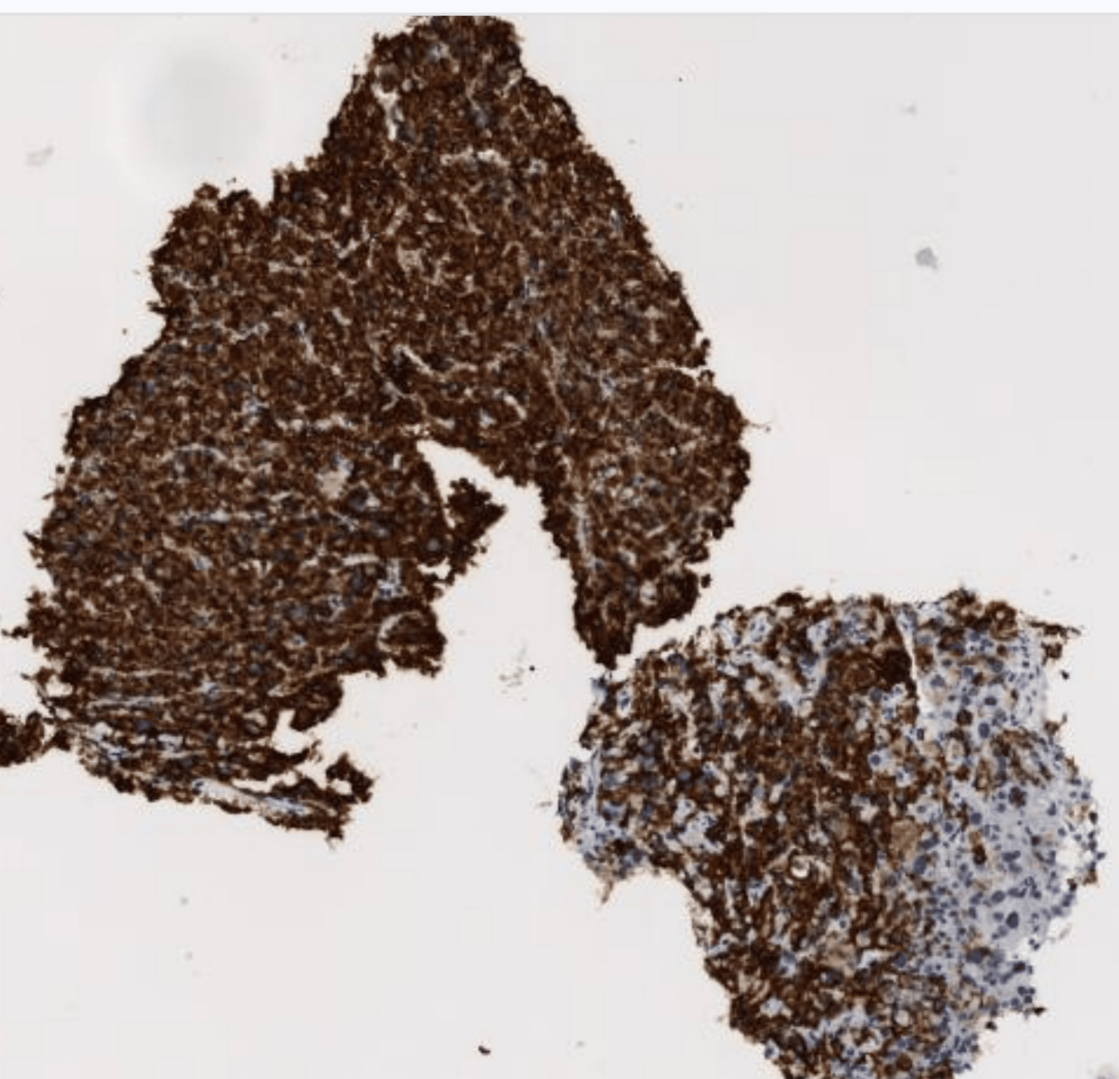
Case 2: IHC showing CD10 diffuse positivity. IHC: immunohistochemical, CD10: cluster of differentiation 10.

**Figure 12:**
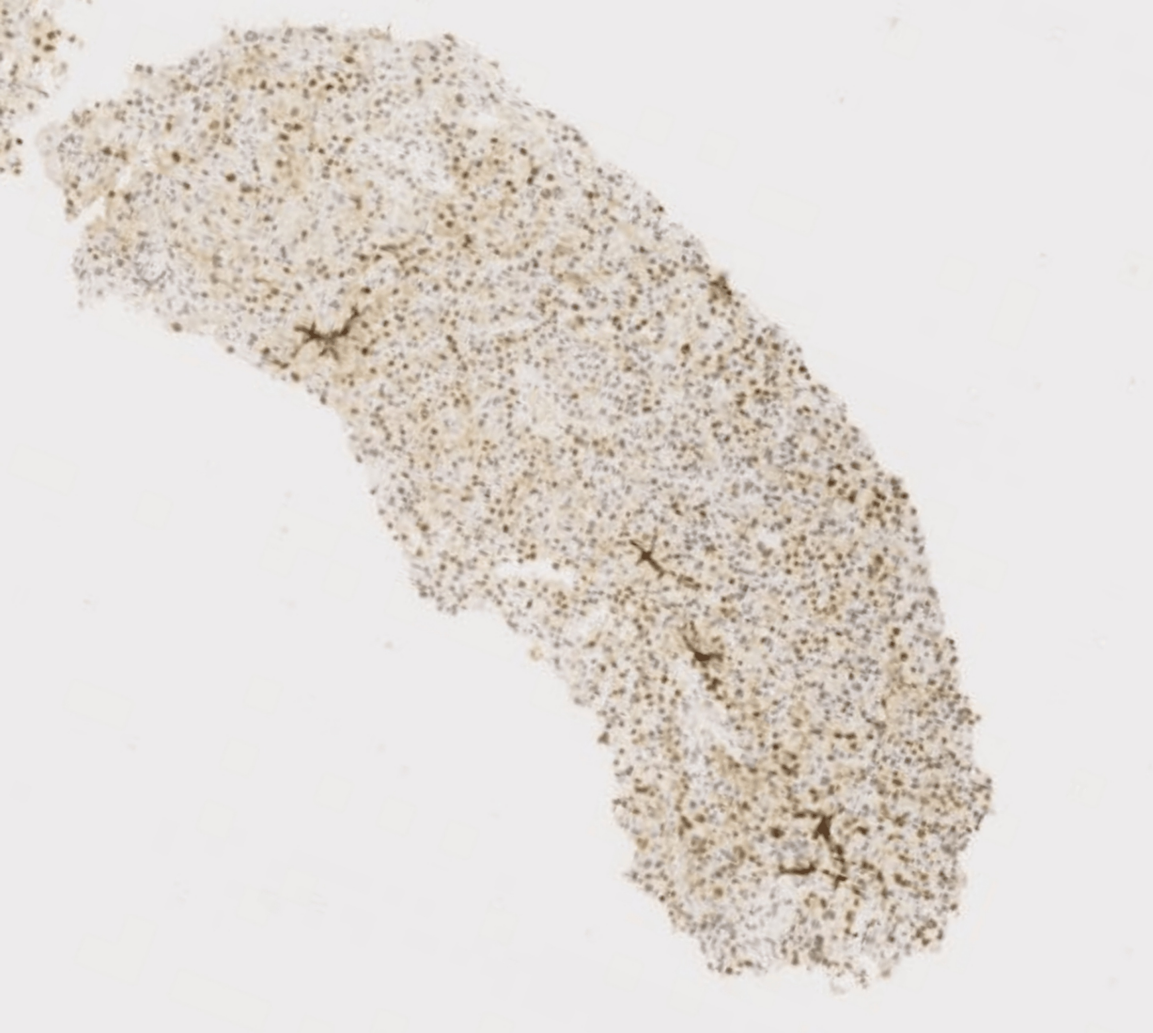
Case 2: IHC showing tumor cell staining nuclear positive for TFE3. IHC: immunohistochemical.

Biopsy revealed a high-grade malignant neoplasm with epithelial features (Figure 10), showing vimentin positivity, diffuse cluster of differentiation 10 (CD10) (Figure 11) and TFE3 (Figure 12) positivity, and carbonic anhydrase IX (CAIX) negativity. In the context of morphology and clinical course, the tumor was classified as TFE3-rearranged RCC, stage IV, with metastatic involvement of the lungs, liver, bones, and adrenal gland (IMDC poor risk, based on anemia (hemoglobin: 6.3 g/dL), poor performance status (KPS: 50), thrombocytosis (platelet count: 510,000/µL), neutrophilia (ANC: 9,000/µL), and time from diagnosis to initiation of systemic therapy <1 year, with no evidence of hypercalcemia (corrected calcium: 9.2 mg/dL)).

During the disease course, she developed an extradural spinal lesion at C4-C5 leading to grade 3 cord compression. She received palliative external beam radiotherapy (20 Gy in 5 fractions) to the cervical spine for metastatic epidural cord compression, along with systemic therapy with Cabozantinib (60 mg once daily). Despite treatment, her disease progressed clinically without formal radiologic assessment per Response Evaluation Criteria in Solid Tumors (RECIST) criteria and she died in January 2025.

Case 3

**Figure 13:**
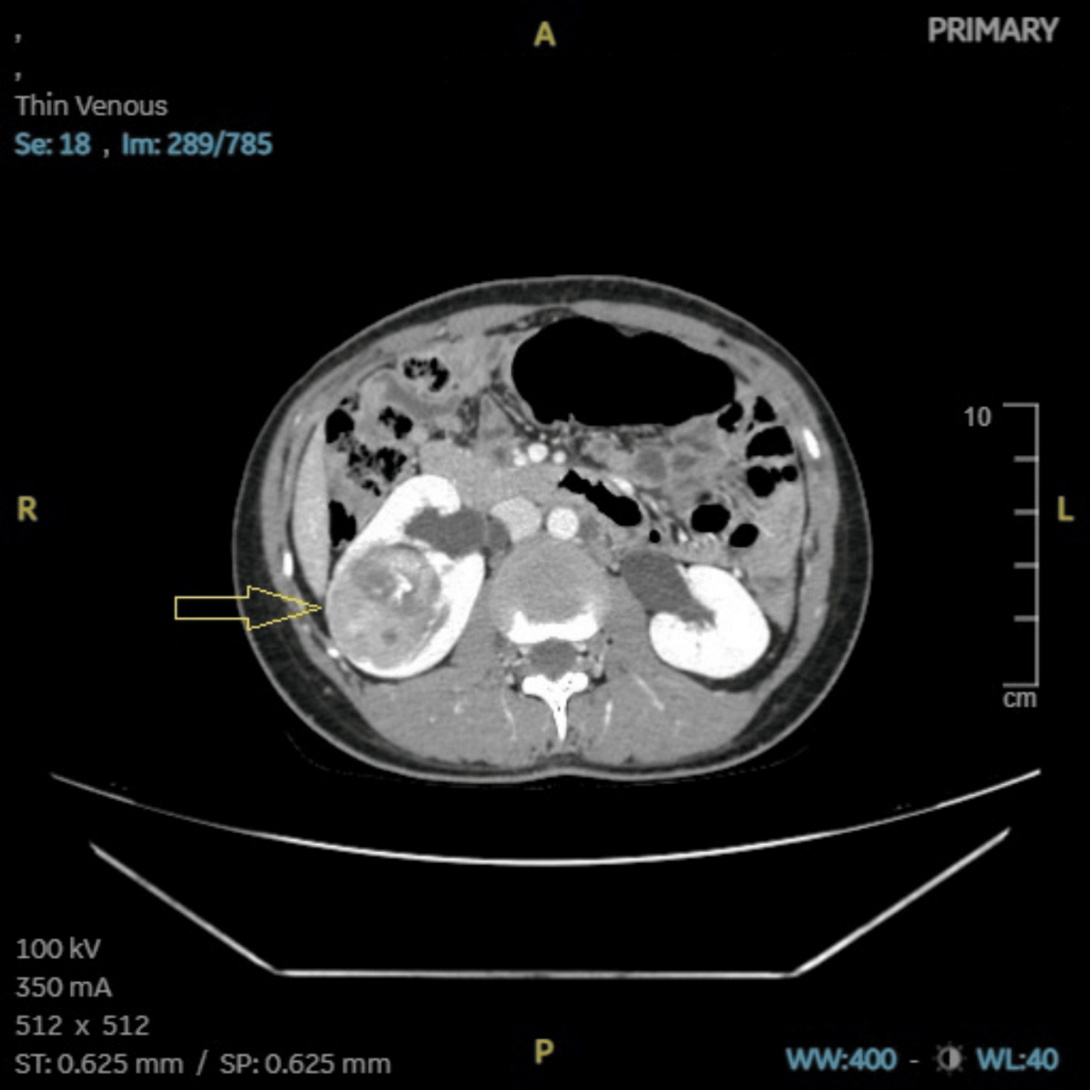
Case 3: CECT (abdomen and pelvis), right renal mass measuring 4.4 × 3.6 × 5 cm (arrow). CECT: contrast-enhanced computed tomography, A: anterior, R: right, L: left, P: posterior, ST: soft tissue, SP: soft tissue window (preset), WW: window width, WL: window level.

**Figure 14:**
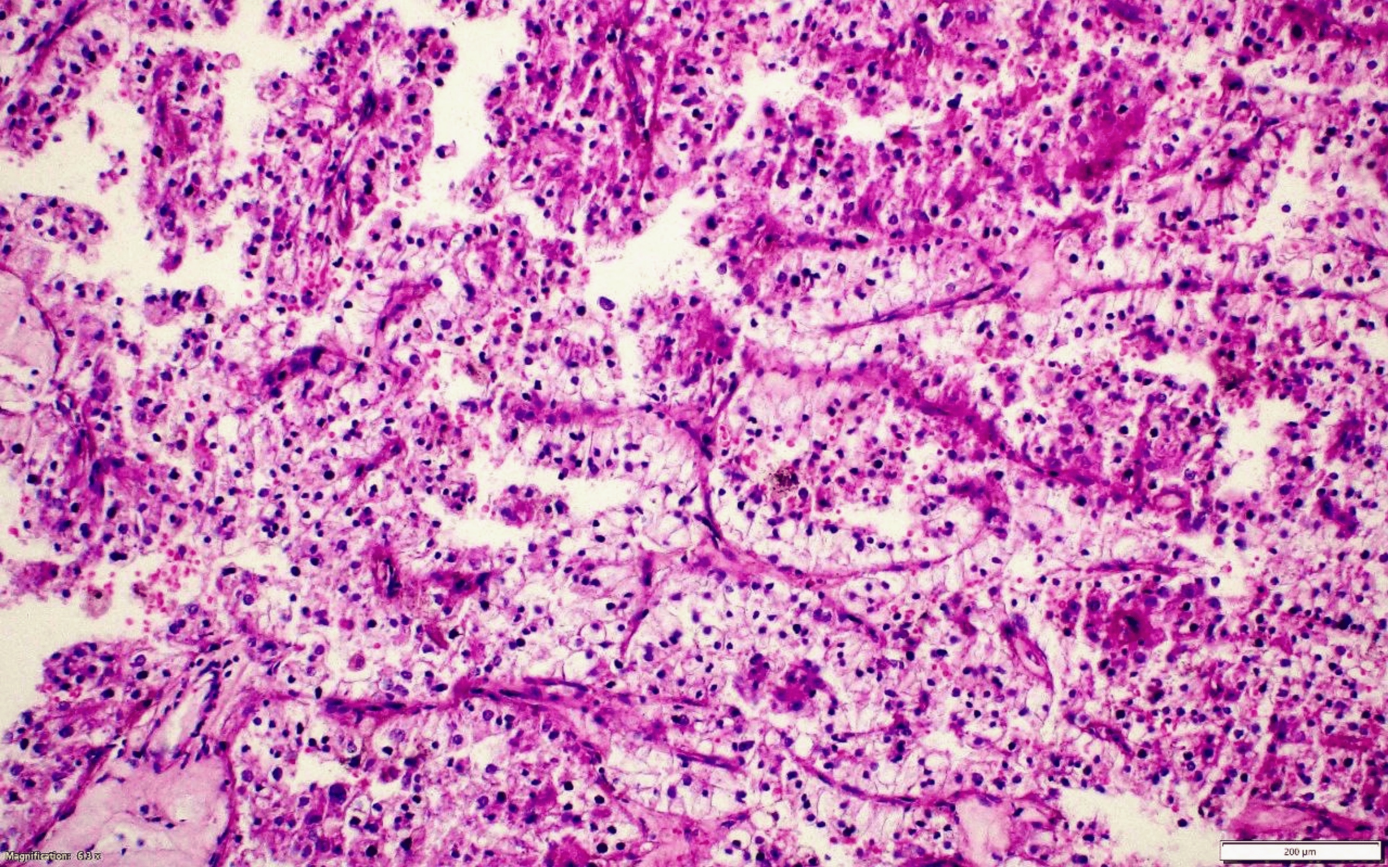
Case 3: Postsurgery renal lesion histopathology (HE) tumor cells arranged in papillae with thin fibrovascular core. Individual cells are large and polygonal with a round nucleus and coarse chromatin with abundant clear cytoplasm. HE: hematoxylin and eosin stain.

**Figure 15:**
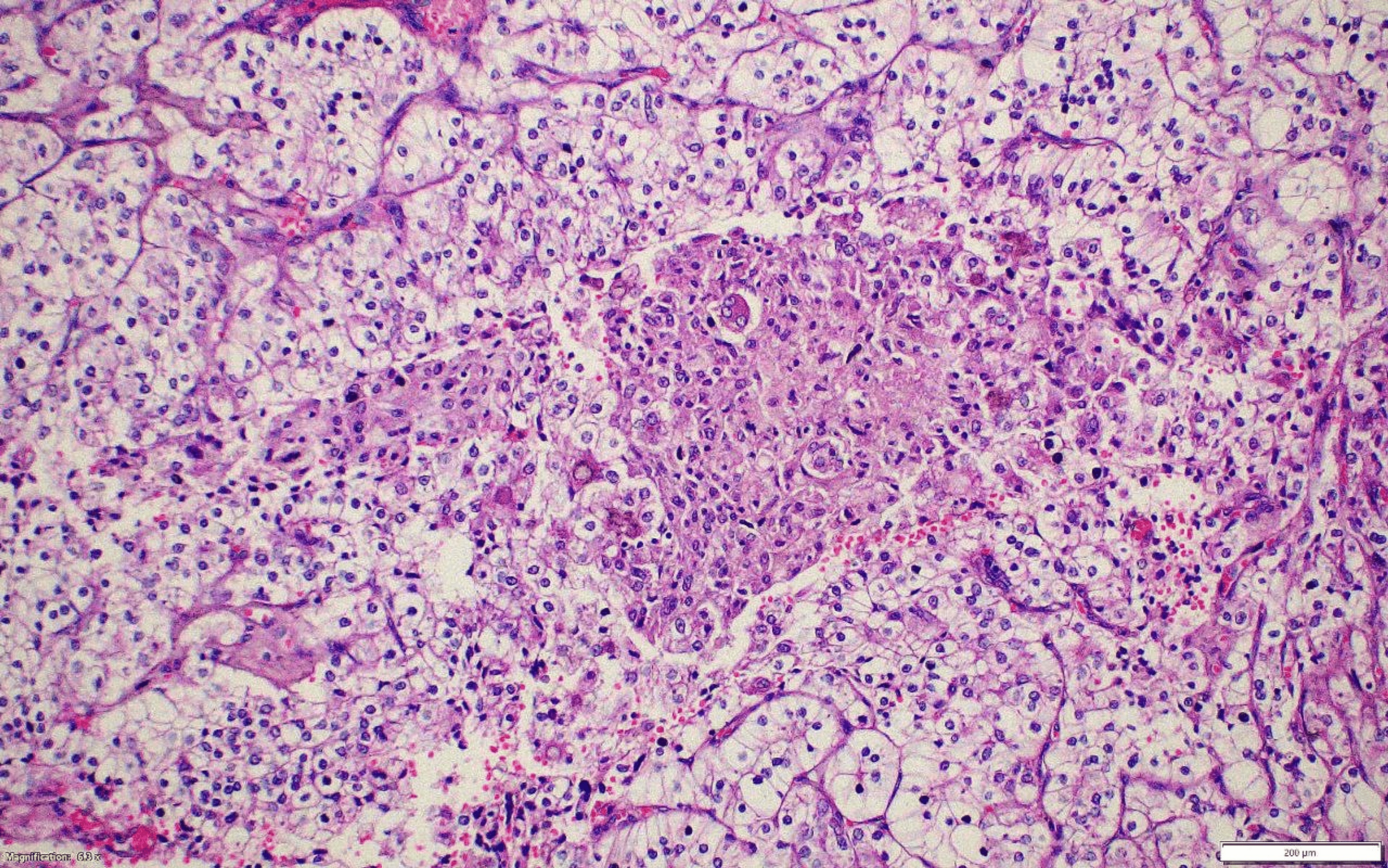
Case 3: Postsurgery renal lesion histopathology (HE) tumor cells with voluminous cytoplasm and discohesive/free floating cell clusters. HE: hematoxylin and eosin stain.

**Figure 16:**
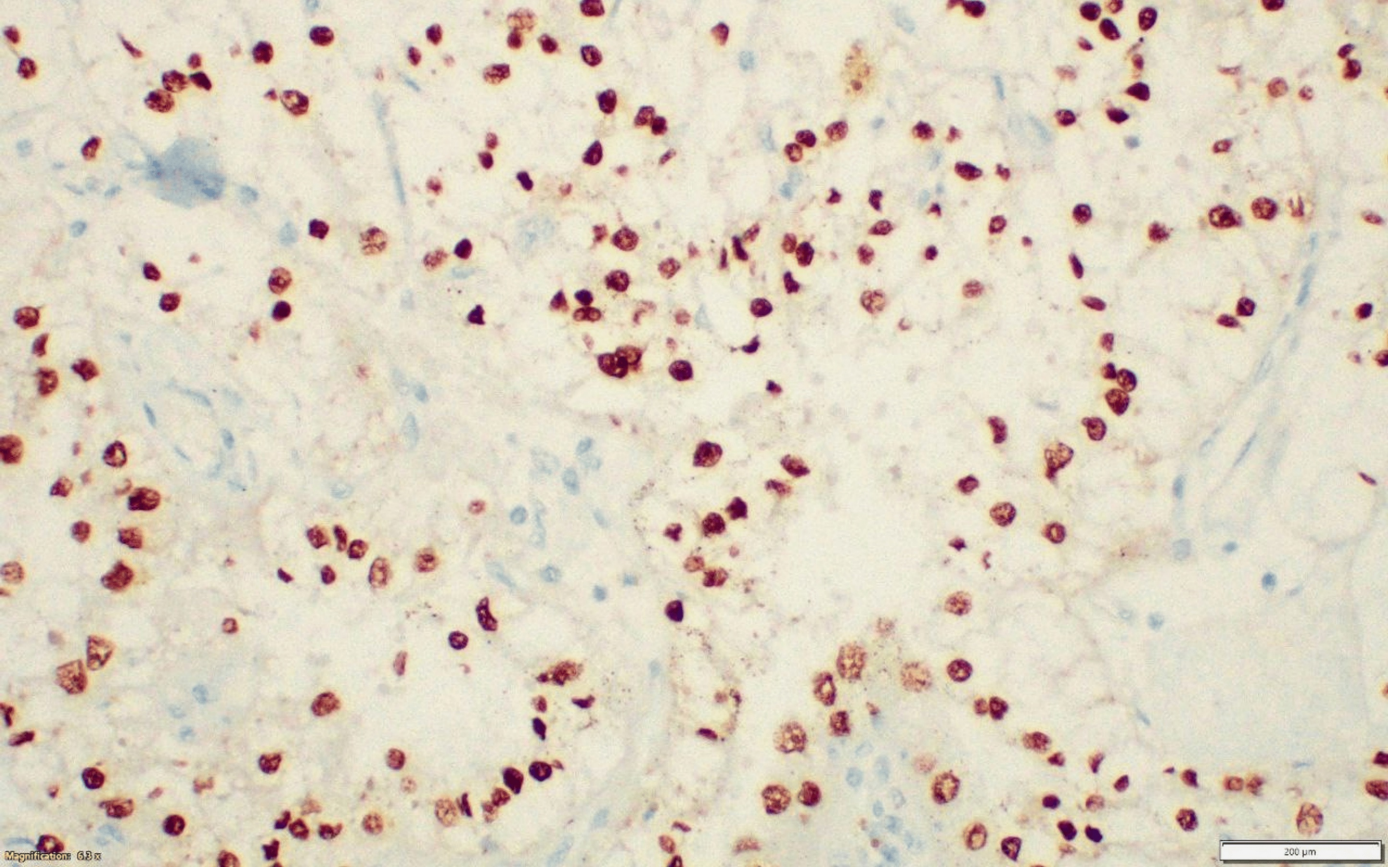
Case 3: IHC tumor cells strong positivity for TFE3. IHC: immunohistochemical.

**Figure 17:**
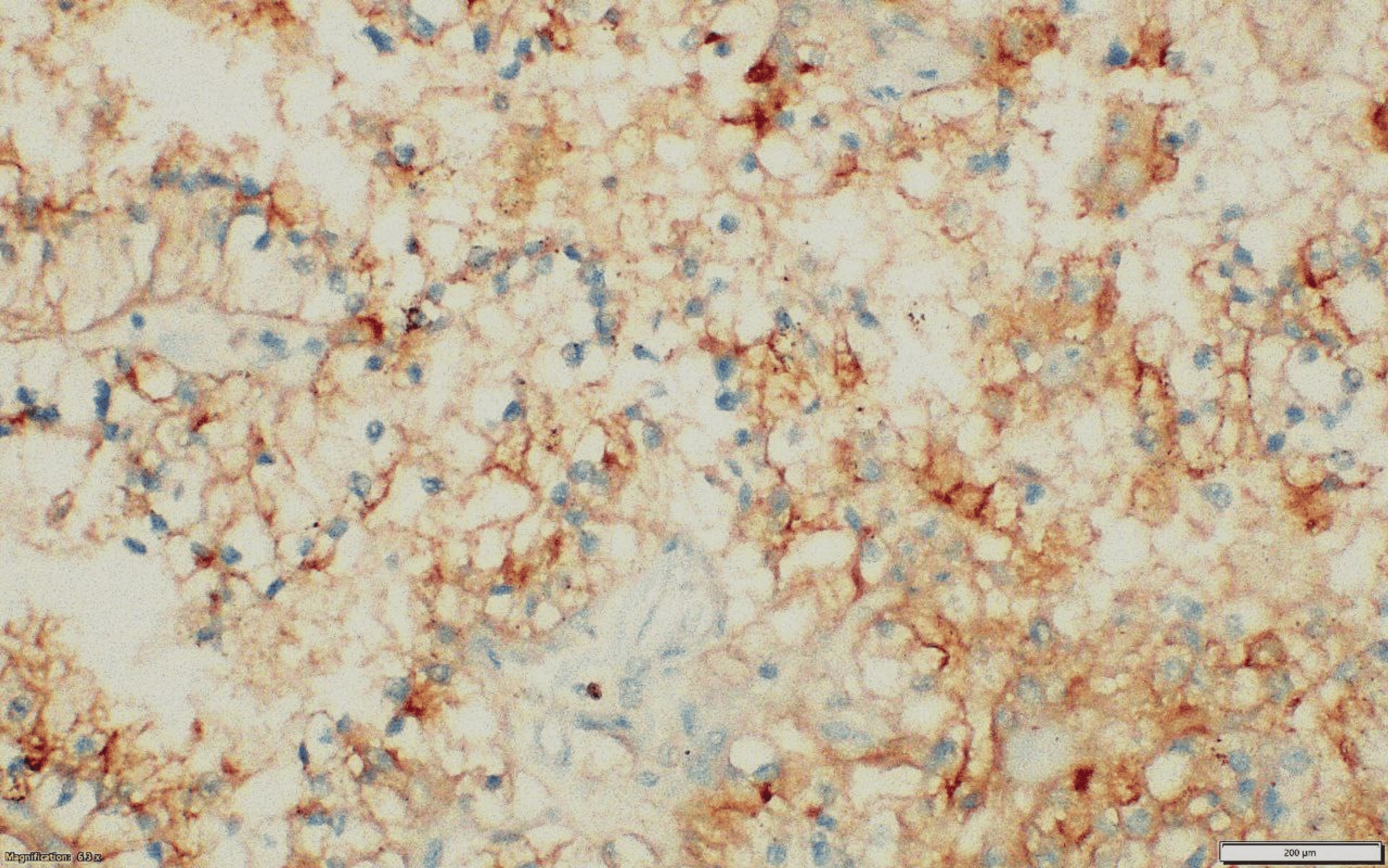
Case 3: IHC tumor cells strong positivity for CD10. IHC: immunohistochemical, CD10: cluster of differentiation 10.

A 22-year-old woman was evaluated for a right renal mass measuring 4.4 × 3.6 × 5 cm (Figure 13). There was radiologic evidence of metastatic disease in the form of a right hilar lymph node. She underwent partial nephrectomy. Histology showed a papillary architecture with clear cytoplasm and capsular and vascular invasion (Figures 14, 15). IHC revealed strong positivity for TFE3 (Figure 16) and CD10 (Figure 17), confirming TFE3-RCC, stage III (pT1N1M0). At one-year follow-up after surgery, she remained disease-free without adjuvant systemic therapy, consistent with more favorable outcomes reported in a study by Liu et al., where 73.5% presented with early stage disease with a three-year overall survival of 100% [11].

Case 4

**Figure 18:**
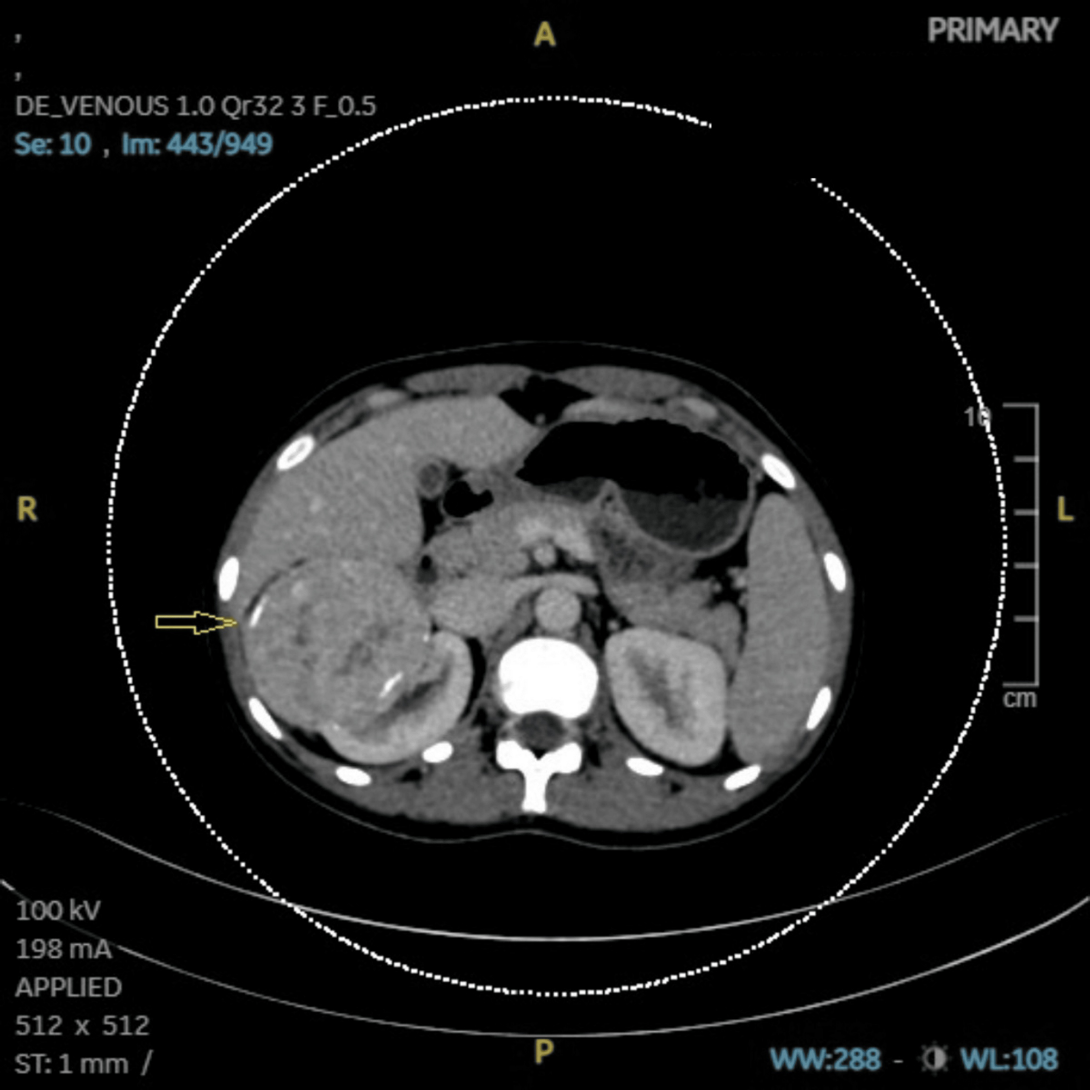
Case 4: CECT (abdomen and pelvis) showing large (9.5 × 7 × 6 cm) right renal mass with peripheral curvilinear calcification (arrow). CECT: contrast-enhanced computed tomography, A: anterior, R: right, L: left, P: posterior, ST: soft tissue, WW: window width, WL: window level.

Another 22-year-old woman presented with a large right renal mass measuring 9.5 × 7 × 6 cm with peripheral curvilinear calcification on CT (Figure 18). Grossly and microscopically, the tumor exhibited unusual coarse brown-black melanin pigmentation, raising a strong differential diagnosis of PEComa.

She underwent radical nephrectomy. Histopathology revealed a tumor with features consistent with TFE3-RCC, showing strong nuclear TFE3 immunopositivity and negativity for cytokeratin, paired box gene 8 (PAX8), and CA9. Although melanocytic markers (human melanoma black 45 (HMB45)/Melan-A) showed positivity, the diagnosis favored TFE3-rearranged RCC over PEComa based on characteristic morphology and diffuse nuclear TFE3 expression. The final stage was stage III (pT3aN0M0). The melanotic differentiation posed a significant diagnostic challenge requiring a broad IHC panel to exclude PEComa and conventional RCC. At six-month follow-up, she remained free of disease (Table 1).

**Table 1:** TFE3-related RCC patients, their characteristics, clinicopathological features, and outcome. RCC: renal cell carcinoma, IMDC: International Metastatic RCC Database Consortium, EBRT: external beam radiotherapy, RT: radiotherapy.

Feature	Case 1	Case 2	Case 3	Case 4
Age / Sex	51 / Female	43 / Female	22 / Female	22 / Female
Presenting symptoms	Left leg pain and swelling	Abdominal pain	Abdominal distension, dysmenorrhea	Fever, abdominal pain, chest pain
Tumor side	Left	Right	Right	Right
Tumor size	Post‑nephrectomy (details unavailable)	6.5 × 5.5 × 6 cm	4.4 × 3.6 × 5 cm	9.5 × 7 × 6 cm
Histopathology	Epithelioid, clear cell, and oncocytic features	High‑grade malignant neoplasm with epithelial features	Papillary architecture, clear cytoplasm	Melanotic pigment, rhabdoid features
Immunohistochemistry	TFE3+, Melan‑A+, HMB‑45+	TFE3+, CD10+, Vimentin+	TFE3+, CD10+	TFE3+, HMB‑45+, Melan‑A+
Pathological stage	Stage IV IMDC-Poor risk	Stage IV IMDC-Poor risk	Stage III (pT3aN1M0)	Stage III (pT3aN0M0)
Metastatic sites	Retroperitoneal and iliac nodes, liver	Lung, liver, adrenal, bone (C5, iliac)	Right hilar lymph node	None
Primary treatment	Radical nephrectomy (2018)->EBRT+Sunitinib	Cabozantinib + palliative spinal RT	Right partial nephrectomy	Right radical nephrectomy
Systemic therapy	Cabozantinib (August 2025 to present)	Cabozantinib	None	None
Outcome/Follow‑up	On treatment, clinically stable	Deceased (January 2025)	Disease‑free at 1 year	Disease‑free at 6 months

This case aligns with rare reports by Jing et al. on melanotic Xp11 translocation renal cancer as a rare and distinctive variant characterized by melanin pigmentation, TFE3 rearrangement, and overlapping features with MiT family translocation tumors, emphasizing the importance of molecular confirmation to avoid diagnostic pitfalls [13].

## Discussion

Epidemiology and classification

The new WHO 2022 classification of RCC introduced molecularly defined RCC categories and formally separated TFE3-rearranged RCC from TFEB-altered RCC [6]. TFE3-RCC is uncommon, representing roughly 1%-4% of RCC overall, but a larger fraction of pediatric and adolescent RCC [7-9]. In a 34-patient cohort, stage I/II disease demonstrated 100% three-year overall survival, whereas advanced Tumor, Node, Metastasis (TNM) stage and inferior vena cava tumor thrombosis independently predicted poor progression-free survival [11]. Multicenter data further suggest more aggressive behavior and inferior outcomes in adults despite higher lymph node positivity in pediatric cases. Our series reflects this spectrum: two young women with localized disease and good early outcomes, contrasted against two adult women with stage IV disease and a poor prognosis [10]. Notably, all patients in our series were female; while this may reflect sampling variation given the very small cohort, it remains an interesting observation.

Diagnostic challenges and role of immunohistochemistry

Morphologically, TFE3-RCC may mimic papillary RCC, clear cell RCC, or PEComa, especially in tumors with clear cytoplasm or granular eosinophilic cells [3]. In our first case, the primary tumor had been reported as clear cell RCC, with the TFE3 nature only identified upon metastatic recurrence, echoing published observations that TFE3-RCC may be under-recognized and misclassified. Case 4 illustrates the challenge posed by melanotic differentiation, with prominent brown-black pigment and positivity for melanocytic markers leading to overlap with PEComa. Such melanotic TFE3-RCC variants have been rarely reported and may require a combination of TFE3 IHC and molecular testing to distinguish them from PEComa and other pigmented renal tumors [13,16,17].

Thus, a comprehensive IHC panel including TFE3, PAX8, CA9, cytokeratins, CD10, HMB45, and Melan-A is critical in problematic cases, and equivocal TFE3 staining should prompt consideration of fluorescence in situ hybridization (FISH) or molecular confirmation, especially in younger patients. However, TFE3 immunohistochemistry alone is not entirely specific, and molecular confirmation by break-apart FISH or RNA-based sequencing is preferable whenever feasible, particularly in tumors with melanotic or PEComa-like features.

Clinical behavior and outcomes

Our series underscores the heterogeneous clinical course of TFE3-RCC. Localized stage III tumors (cases 3 and 4) showed no recurrence at short-term follow-up after surgery alone, consistent with good outcomes in early-stage TFE3-RCC reported in some series [10,11]. However, the short follow-up duration limits interpretation of durable disease control in these localized cases.

In contrast, case 2 illustrates the aggressive potential of metastatic TFE3-RCC with rapid progression and spinal cord compression, in line with studies documenting high rates of metastasis and reduced survival in advanced disease [12,15,18,19]. Case 1 highlights a unique aspect of TFE3-RCC, late recurrence after years of apparent control on targeted therapy, emphasizing the need for prolonged surveillance even after initial curative-intent treatment.

Systemic therapy

For patients without metastasis, surgery is the first-line treatment, whereas targeted therapies such as vascular endothelial growth factor receptor tyrosine kinase inhibitors (VEGFR-TKIs) (e.g., sunitinib, sorafenib, cabozantinib) and more recently immune checkpoint inhibitors have been used in metastatic TFE3-RCC with variable efficacy [15,20]. More recent series suggest that TKI- and immunotherapy-based regimens can yield meaningful responses, although outcomes remain inferior to those of typical clear cell RCC [18]. Malouf et al. reported that targeted therapies in Xp11 translocation RCC achieved objective responses and progression-free survival comparable to those seen in clear cell RCC [20].

In our cohort, patients with advanced disease (cases 1 and 2) received targeted therapy; one experienced a prolonged clinically stable phase followed by late recurrence, while the other demonstrated clinical progression despite treatment with Cabozantinib and palliative radiotherapy. This divergence illustrates the unpredictable therapeutic response and the need for more robust evidence to guide systemic treatment in TFE3-RCC.

Strengths and limitations

Strengths of this series include detailed clinicopathologic characterization of a rare entity and documentation of a melanotic TFE3-RCC variant in a young adult, from an Indian tertiary cancer center, adding to the global literature. Limitations include the small sample size, retrospective nature, short follow-up for localized cases, and lack of molecular confirmation (e.g., FISH or next-generation sequencing) in all cases due to financial constraints, which is common in resource-limited settings. Additional limitations include lack of standardized RECIST-based response assessment and limited follow-up for localized disease.

## Conclusions

TFE3-rearranged RCC is a rare but clinically important subtype that often affects younger patients and may present with advanced disease and aggressive behavior. Our case series demonstrates diagnostic complexity, particularly in tumors with melanotic differentiation mimicking PEComa or with morphology overlapping clear cell or papillary RCC. Strong nuclear TFE3 immunoreactivity, interpreted alongside morphology and a broad immunohistochemical panel, is central to diagnosis; however, molecular confirmation remains desirable wherever feasible. Heterogeneous clinical outcomes range from long-term disease control with late recurrence to rapid progression and death in metastatic cases. The importance of long-term surveillance after nephrectomy and individualized use of targeted and/or immunotherapy-based regimens in advanced disease is emphasized. Greater awareness of this entity, routine consideration of TFE3-RCC in younger patients with atypical RCC morphology, and broader access to molecular diagnostics will improve recognition and management of these tumors. Collaborative registries with integrated molecular characterization and longer follow-up are needed to better define prognosis and optimize systemic therapy in adult TFE3-rearranged RCC.
